# Treatment of Focal Cartilage Defects in Minipigs with Zonal Chondrocyte/Mesenchymal Progenitor Cell Constructs

**DOI:** 10.3390/ijms20030653

**Published:** 2019-02-02

**Authors:** Friederike Bothe, Anne-Kathrin Deubel, Eliane Hesse, Benedict Lotz, Jürgen Groll, Carsten Werner, Wiltrud Richter, Sebastien Hagmann

**Affiliations:** 1 Research Center for Experimental Orthopaedics, Heidelberg University Hospital, Germany, Schlierbacher Landstr. 200a, 69118 Heidelberg, Germany; friederike.bothe@med.uni-tuebingen.de (F.B.); anneknauf@mailbox.org (A.-K.D.); Eliane.Hesse@med.uni-heidelberg.de (E.H.); Wiltrud.Richter@med.uni-heidelberg.de (W.R.); 2 Center of Orthopaedic and Trauma Surgery/Spinal Cord Injury Center, Heidelberg University Hospital, Germany, Schlierbacher Landstr. 200a, 69118 Heidelberg, Germany; Benedict.Lotz@med.uni-heidelberg.de; 3 Department of Functional Materials in Medicine and Dentistry and Bavarian Polymer Institute, University of Würzburg, Pleicherwall 2, 97080 Würzburg, Germany; carsten.werner@tu-dresden.de; 4 Leibniz Institute of Polymer Research Dresden, Max Bergmann Center of Biomaterials Dresden, 01069 Dresden, Germany; carsten.werner@tu-dresden.de

**Keywords:** cartilage repair, osteochondral defect, tissue engineering, starPEG hydrogel, chondrocyte, MSC, zonal construct, minipig

## Abstract

Despite advances in cartilage repair strategies, treatment of focal chondral lesions remains an important challenge to prevent osteoarthritis. Articular cartilage is organized into several layers and lack of zonal organization of current grafts is held responsible for insufficient biomechanical and biochemical quality of repair-tissue. The aim was to develop a zonal approach for cartilage regeneration to determine whether the outcome can be improved compared to a non-zonal strategy. Hydrogel-filled polycaprolactone (PCL)-constructs with a chondrocyte-seeded upper-layer deemed to induce hyaline cartilage and a mesenchymal stromal cell (MSC)-containing bottom-layer deemed to induce calcified cartilage were compared to chondrocyte-based non-zonal grafts in a minipig model. Grafts showed comparable hardness at implantation and did not cause visible signs of inflammation. After 6 months, X-ray microtomography (µCT)-analysis revealed significant bone-loss in both treatment groups compared to empty controls. PCL-enforcement and some hydrogel-remnants were retained in all defects, but most implants were pressed into the subchondral bone. Despite important heterogeneities, both treatments reached a significantly lower modified O’Driscoll-score compared to empty controls. Thus, PCL may have induced bone-erosion during joint loading and misplacement of grafts in vivo precluding adequate permanent orientation of zones compared to surrounding native cartilage.

## 1. Introduction

Despite the medical and socioeconomic burden of osteoarthritis [1], there is still no cure available for the disease. Although some risk factors for osteoarthritis have been identified [2], it is largely accepted that the disease remains at a subclinical level for some time and that once joint destruction has started, it cannot be reversed. Some treatments seem to slow down the process fueling joint degeneration, but there is no alternative to joint replacement in the ultimate late-stages of osteoarthritis. Another challenge for medicine is the treatment of chondral and osteochondral lesions in younger patients, which are believed to ultimately develop into osteoarthritis as well.

It is, therefore, indispensable to develop alternative therapies for osteoarthritis and (osteo-) chondral defects. An important approach is to recreate the destroyed tissue by in vitro engineering. The most important pillars of tissue engineering (TE) are the use of cells, biomaterials, and growth factors. With the establishment of autologous chondrocyte transplantation, chondrocytes are the most prominent cell type to be used in tissue engineering [3]. Another promising cell types are mesenchymal stromal cells (MSC). Not only can MSC be derived from numerous tissues and easily expanded in vitro, they also bear an important potential to differentiate into several directions, such as chondrogenic, osteogenic, and adipogenic lineages. These abilities have made MSCs one of the most important cells for tissue engineering (overview in [4]), although their clinical use is not as widespread as that of chondrocytes.

Numerous TE therapies have been developed for the treatment of chondral and osteochondral defects, the most important being autologous chondrocyte implantation [5], which has evolved towards matrix-associated cell-based technique (MACT or MACI, [6]). Numerous approaches modifying the biomechanical properties and the composition of the biomaterials (overview in [7,8,9]) have been proposed to facilitate the regeneration of the defect. 

Despite research focusing on tissue regeneration, and cartilage regeneration over the past decade, the recreation of joint-like hyaline cartilage has not been obtained. One of the reasons may be that articular cartilage has a complex microstructure involving an important vertical organization of chondrocytes. According to their zonal position, chondrocytes seem to exert distinct properties, as has been suggested after the isolation of chondrocytes from different layers [10]. Most of the studies involving scaffolds for cartilage regeneration did not involve recreating its zonal design. An interesting approach, therefore, is to mimic the ultrastructure of cartilage by introducing two- or multilayered scaffold designs [11]. The use of multiple materials and the zonal distribution of specific cells in this regard is sometimes referred to as biofabrication [12].

Several studies suggest that two- or multilayered constructs facilitate integration into the defects and improve overall regeneration. For instance, biphasic cartilage-ceramic constructs showed important improvement of regeneration in osteochondral defects in sheep [13]. In a caprine model, a multilayered biomimetic collagen-based scaffold showed improved healing of osteochondral defects when compared to empty and market approved scaffolds [14]. The same group was able to demonstrate that a multilayered construct resulted in zonal tissue regeneration, involving subchondral bone, the cartilaginous layer, and an intermediate tidemark [15]. Guo et al. reported a full restoration of cartilage and subchondral bone in osteochondral defects in rabbits when applying poly(lactid-co-glycolid)/articular cartilage extracellular matrix PLGA/ACECM composite scaffolds to the defects [16]. Most importantly, the authors claim that they observed the development of hyaline cartilage organized in a structure similar to native tissue. 

Based on these findings, we aimed at determining whether hydrogel-filled polycaprolactone (PCL)-constructs with a chondrocyte-seeded upper-layer and an MSC-seeded bottom-layer deemed to induce calcified cartilage could improve cartilage regeneration of superficial osteochondral defects in vivo. We, therefore, compared these constructs to a PCL-non-zonal construct as well as to empty defects in a minipig model.

## 2. Results

### 2.1. Similar Absolute Hardness of Zonal and Non-Zonal Constructs Before Implantation 

**Figure 1:**
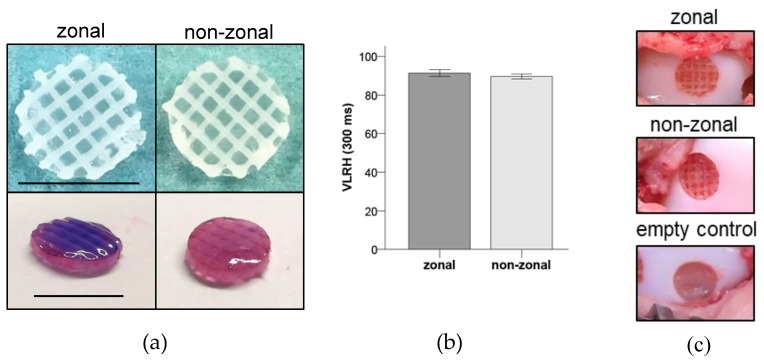
Design of enforced constructs and biomechanical properties. (**a**) The polycaprolactone (PCL) enforcement was soaked with 30 µL cell-containing starPEG hydrogel in a zonal (15 µL + 15 µL) or non-zonal design. Top: Top view on constructs ready for implantation. Bottom: Dark violet zone represents the mesenchymal stromal cell (MSC)-containing bottom zone of the zonal construct, light violet the chondrocyte-containing zone of both constructs. Scale bar = 6 mm. (**b**) Evaluation of the absolute hardness values very low rubber hardness (VLRH) of zonal and non-zonal implants. Mean ± SD. *n* = 5 per group. (**c**) Representative pictures of defects treated with zonal or non-zonal implants and the empty control directly before wound closure.

To determine the absolute hardness values at the time of implantation, zonal and non-zonal constructs (Figure 1a) were measured directly after construct preparation. No significant differences were obtained between zonal and non-zonal groups indicating the same biomechanical properties at the time of implantation into minipig knees (Figure 1b).

### 2.2. Inferior Gross Appearance and Substantial Subchondral Bone Changes in Implant-Treated Defects 

**Figure 2:**
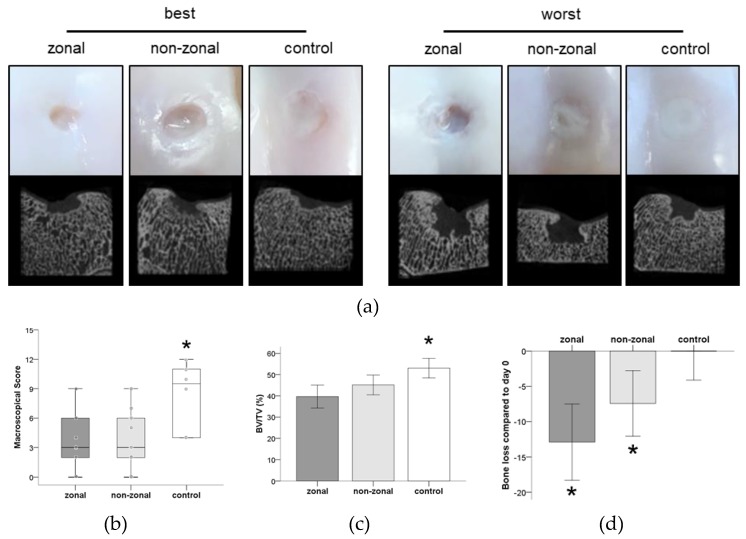
Macroscopy and X-ray microtomography (µCT) of treated defects after 6 months in vivo. (**a**) Shown are the best and worst µCT images and the corresponding macroscopic appearance per group. (**b**) International Cartilage Repair Society (ICRS) score determined by three blinded observers resulted in a significantly better score of empty defects compared to both treatment groups. Boxes represent first and third quartiles, medians are given as horizontal lines, whiskers are maximal and minimal values, individual values (mean of all three observers) are depicted as circles. *: *p* ≤ 0.05 vs. other groups (Mann–Whitney-U test (MWU), Bonferroni correction). Zonal *n* = 9, non-zonal *n* = 9, control *n* = 6. (**c**) Quantification of bone volume/total volume 6 months after treatment. Mean ± SD. *: *p* ≤ 0.05 vs. other groups (ANOVA, Bonferroni correction). Zonal *n* = 9, non-zonal *n* = 9, control *n* = 6. (**d**) Bone loss after treatment with zonal and non-zonal PCL-enforced starPEG constructs and in empty control defects over 6 months. Mean percentage of bone loss ± SD per group compared to day 0 defects (*n* = 4). *: *p* ≤ 0.05 vs. control (ANOVA, Bonferroni correction). Zonal *n* = 9, non-zonal *n* = 9, control *n* = 6.

At study termination six months after surgery, no signs of inflammation or intra-articular pathological changes were observed in the joints. Macroscopy revealed visible PCL enforcement in 5/18 defects. In three other defects, PCL was suspected macroscopically. No signs of deterioration were seen in these visible enforcements. All empty defects were filled with white tissue. Most of the PCL-treated defects displayed similar white tissue, but mainly at the edge of the defects and not across the center. In general, defects of both treatment groups had a more concave surface whereas empty controls were almost at level with the surrounding cartilage (Figure 2a). Repair quality was rated by macroscopic evaluation by three independent observers and revealed significantly better results for the empty control defects. No significant differences were found between the zonal and non-zonal group (Figure 2b).

Micro-CT visualization revealed bone damage in all three groups, with a similar degree of variability between different defects (Figure 2a). Bone volume/total volume (BV/TV) was quantified in a standardized volume of interest for each defect. Mean BV/TV was 39.66% for the zonal group (26.33–44.09%), 45.14% for the non-zonal group (38.66–54.06%), and 53.07% for empty controls (48.60–60.12%) (Figure 2c). Thus, the defects of the empty control group contained significantly more mineralized tissue compared to defects which had received implants. The non-zonal group showed a trend to more bone retention than zonal defects (*p* = 0.085; Figure 2c). 

Compared to freshly prepared defects (day 0, *n* = 4), the mean BV/TV decreased by 12.91% in the zonal group, by 7.43% in the non-zonal group, and increased by 0.50% in the empty controls (Figure 2d). Thus, PCL-treated defects lost significantly more bone (zonal *p* = 0.000, non-zonal *p* = 0.019) than control defects indicating an osteolytic effect of PCL-enforced implants on the subchondral bone (Figure 2d). In summary, treatment with PCL-enforced constructs, independent of a zonal or non-zonal hydrogel organization, resulted in more bone erosion and a worse macroscopic appearance than leaving the defects untreated.

### 2.3. Implant Retention and Dislocation into Subchondral Bone

**Figure 3:**
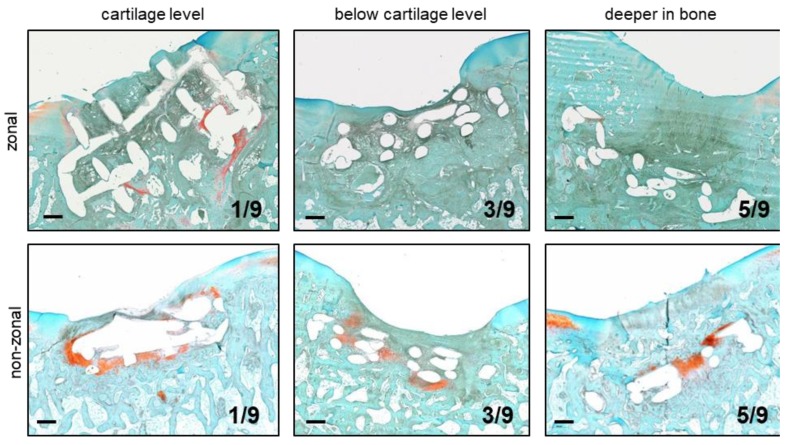
Implant location after 6 months in vivo according to the histology of cartilage defects. First column: Implant with the location of PCL surface at cartilage level. Second column: Implant with the location of PCL surface ≤1-fold construct thickness below cartilage surface. Third column: Implant with the location of PCL surface >1-fold construct thickness below cartilage surface. Numbers: constructs/group. Safranin O/Fast Green staining. Zonal, non-zonal *n* = 9. Representative pictures are shown in the middle and right columns.

Histology demonstrated that the enforcement was retained in all 18 PCL-treated defects, though single constructs appeared inclined to the defect presenting a greater section of the PCL enforcement than the expected cross-section (Figure 3, zonal at cartilage level). However, in 16/18 lesions the PCL was pressed below cartilage level into the subchondral bone. Often cancellous bone began to grow into the enforcement leading to a seamless tissue integration (Figure 3). Only one construct per group remained at its original position at cartilage level, 3/9 were pressed ≤1-fold construct thickness into the subchondral bone, and 5/9 were found deeper than construct thickness in the underlying bone (Figure 3). In summary, the PCL enforcement was retained over 6 months in the orthotopic environment with little evidence for degradation, but in most cases, it dislocated deeper into the subchondral bone and was, therefore, a potential source for the observed bone lysis. 

### 2.4. Hydrogel Persistence and Cell Differentiation in Zonal Versus Non-Zonal Implants

**Figure 4:**
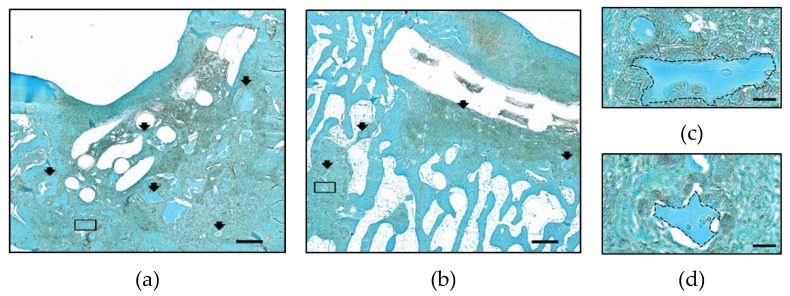
Hydrogel persistence in the zonal and non-zonal groups after 6 months in vivo. Overview of representative defects after treatment with zonal (**a**,**c**) and non-zonal (**b**,**d**) constructs. Black arrows: hydrogel fragments. Rectangles: area of higher magnification shown in (**c**) and (**d**). Hydrogel fragments outside of the PCL enforcement pressed into the subchondral bone indicated by dotted lines. Scale bar: 500 µm (**a**,**b**), 50 µm (**c**,**d**).

Hydrogel fragments were retained in all samples of the zonal and non-zonal group, respectively, but were no longer located only within the former implant. In all samples of the zonal and in 7/9 defects of the non-zonal group residual hydrogel material was pressed into the surrounding tissue and underlying bone (Figure 4a,b). Hydrogel fragments appeared to be in different stages of degradation and, contrary to some hydrogel remnants within the PCL, dislocated fragments were never Safranin-O-positive demonstrating that the sulfation of the heparin-building block had disappeared (Figure 4c,d). However, Safranin-O-positive differentiated areas were found inside some defects of each group with and without construct involvement (Table 1). In the absence of early samples, the time frame during which zonal (versus non-zonal) structure was retained remained unclear.

**Figure 5:**
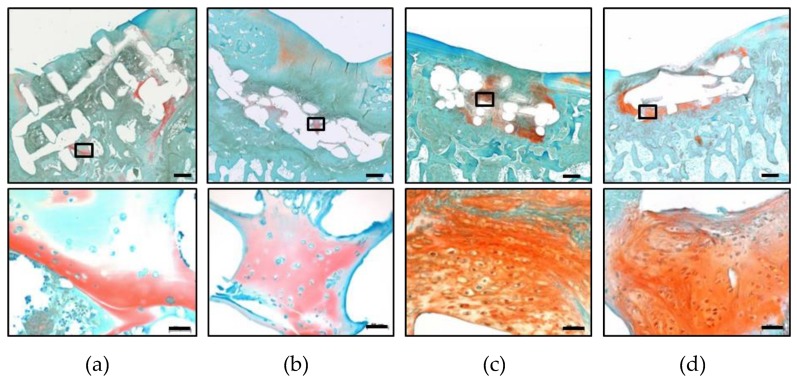
Cell differentiation within the PCL enforcement inside and outside of persisting hydrogel. Best results per group of cells in Safranin-O-positive hydrogel fragments (**a**,**b**) and Safranin-O-positive areas within PCL enforcement with differentiated cells (**c**,**d**) are shown. Rectangle: area of magnification on the bottom. Safranin O/Fast Green staining. Scale bar: 500 µm (overview, top), 50 µm (magnification, bottom).

At six months, most hydrogel fragments outside the PCL enforcement showed no evidence for persistence of grafted cells. In contrast, within the PCL enforcement in two zonal (Figure 5a) and three non-zonal constructs (Figure 5b), Safranin-O-positive cell-containing hydrogel was found indicating that hydrogel and implanted cells may have contributed to the regeneration tissue, although one of these PCL implants appeared not only pressed into the bone but compressed and askew demonstrating yielding of the construct to the acting forces (Figure 5b). In another two defects of each treatment group, Safranin-O-positive areas with differentiated cells were observed in the apparent absence of hydrogel within and connected to the enforcement (Figure 5c,d). Safranin-O-positive host tissue at cartilage level was frequently found in control defects, but also above the enforcement of four PCL-treated defects where the PCL was pressed deeply into the bone (Table 1, Figure 5b). Overall, the Safranin-O-positive regeneration tissue at cartilage level often appeared host-derived and seemed to occur independently of the implant. Only sometimes was the Safranin-O-positive regeneration tissue connected to the transplanted hydrogel. In rare cases, implanted cells persisted in hydrogel fragments and contributed to the Safranin-O-positive regeneration tissue with no differences between the non-zonal chondrocyte design and the zonal MSC/chondrocyte design. 

### 2.5. No Benefit of Zonal or Non-Zonal Design on the Quality of Repair Tissue Compared to Empty Controls

**Figure 6:**
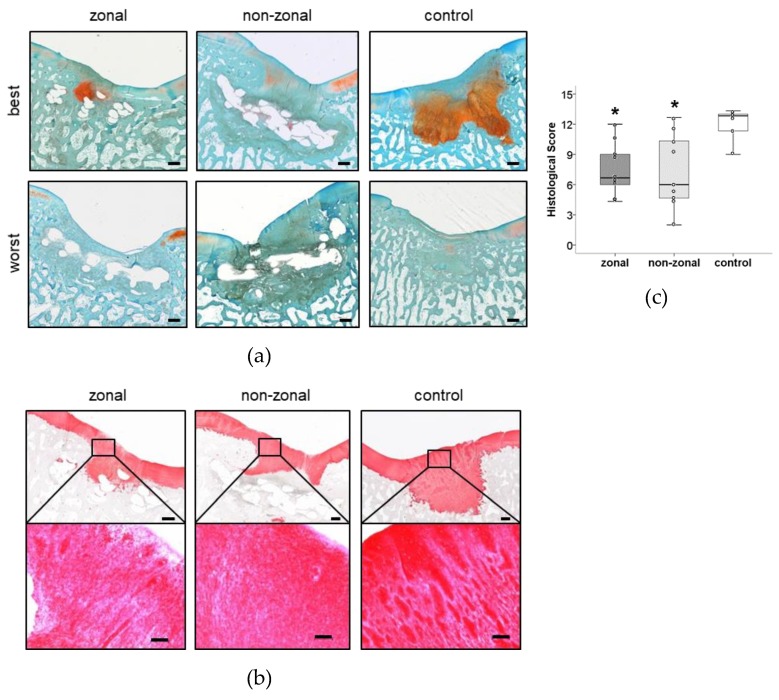
Evaluation by modified O’Driscoll score. (**a**) Best and worst results per group according to histological scoring. Safranin O/Fast Green staining. Scale bar: 500 µm. (**b**) Best cell morphology and collagen type II deposition of regeneration tissue in each group after 6 months in vivo. Top: overview. Scale bar: 500 µm. Bottom: area of magnification. Scale bar: 100 µm. (**c**) Histological evaluation of defect repair after 6 months in vivo rated by three blinded observers using a modified O’Driscoll score. Boxes represent first and third quartiles, medians are given as horizontal lines, whiskers are maximal and minimal values, individual values (mean of all three observers) are depicted as circles. *: *p* ≤ 0.05 vs. control (MWU, Bonferroni correction). Zonal *n* = 9, non-zonal *n* = 9, control *n* = 6.

Defects treated with hydrogel constructs were filled mostly with fibrous regeneration tissue with some proteoglycan-positive spots around cartilage level and within the PCL enforcement and more pronounced subchondral bone changes than empty controls. Many defects seemed filled with a similar but less differentiated fibrous regeneration tissue like that seen in empty controls at cartilage level. The best and worst samples per group according to histological scoring are shown in Figure 6. The best defects contained cell-rich repair tissue rich in proteoglycans (Figure 6a) and collagen type II (Figure 6b). Overall, cartilage regeneration graded by a modified O´Driscoll score to rate the nature of the predominant tissue and the structural characteristics was more heterogeneous in the implant-treated groups. Scores ranged from 9 to 13 for empty controls, 4 to 12 for the zonal group, and 2 to 13 for the non-zonal group (Figure 6c). Zonal (*p* = 0.007) and non-zonal groups (*p* = 0.019) reached significantly lower scores than empty control defects, and no specific differences were observed between zonal and non-zonal implant design.

## 3. Discussion

Although attempts at recreating articular cartilage have been made for several decades, so far it has not been achieved to fully restore the unique properties of hyaline cartilage in vivo. Zonal designs mimicking the vertical organization of cells and matrix in a joint are promising tools to enhance cartilage regeneration. Several studies have shown that not only are such designs technically feasible [17,18] but that they can improve integration into osteochondral defects [14,19,20]. The importance of the calcified cartilage has already been depicted in 1975 [21]. It plays a major role in connecting the cartilage to the subchondral bone [22] and enhances shear strength [23]. Yet, most studies focus on restoring the cartilage and the subchondral bone without putting emphasis on the connecting calcified zone.

In this study, we compared a zonal construct consisting of two layers with two different cell types to a non-zonal design for cartilage regeneration. We hypothesized that the zonal approach would facilitate integration [18,24,25,26] into the defects and would result in a more natural tissue when compared to a non-zonal design and to empty control defects. After six months in vivo, zonal and non-zonal constructs showed a significantly increased bone loss in the defects when compared to controls. This bone loss may be because, in most cases, the hydrogel was pressed out of the construct into the underlying bone, suggesting high pressure on the enforcements and, thus, on the bone as well. It can be hypothesized that the stiffness of the constructs may play a role in bone loss and dislocation of the hydrogel into the underlying bone. Allan et al. found in 2007 that the formation of biphasic constructs containing cartilage and designed with a calcified zone interface resulted in better cartilage load-bearing properties and interfacial shear strength [23]. However, this study also demonstrated failure due to shear at the cartilage–calcium–polyphosphate interface, indicating the importance of a solid contact between different biomaterials applied in two- or multilayered constructs. Stiffer constructs seem superior to softer ones regarding cartilage regeneration in vivo [27,28], with good integration into the defects histologically. 

The use of µCT, however, to quantify subchondral bone structure is not widespread and can be considered a strength of this study. The stiffness of both the zonal- and non-zonal constructs at implantation was comparable in our study, which may explain why the amount of bone loss was also comparable in those groups. A comparable issue with PCL-reinforced constructs has been described by Mancini et al. and was partially affiliated to the xenogenic fibrin glue [29]. By using a solid press-fit fixation without glue, we were able to eliminate this variable. The fact that bone loss was observed to a much lower extent in empty defects clearly indicates the influence of pressure on PCL-enforced constructs in this in vivo model, where animals were allowed to fully weight bear immediately after surgery.

The aim of this study was not to repair the subchondral bone, but rather the calcified cartilage layer as well as the hyaline cartilage. Both the zonal and the non-zonal PCL-enforced starPEG constructs were retained in all defects, showing that the primary stability of the constructs was excellent. Also, the printed PCL architecture remained intact, suggesting that the design of the construct allowed for durable stability even in the presence of a full load in the animals. However, in 16 out of 18 defects, the constructs were pressed beneath the cartilage level, which may be attributed to its overall thickness. The thickness of the constructs is determined by their design—multiple layers lead to thicker constructs, and the need for filling these constructs with cells, for example, makes attempts at keeping the constructs flat enough challenging.

Another problem is that with increasing thickness of the constructs, as seen in multilayered scaffolds, the transport of nutrients into the core areas and the transport of waste out of the areas is reduced [30,31]. This leads to a reduced matrix formation in these core areas resulting in decreased biomechanical properties compared to the peripheral zones [32,33]. Several techniques to improve the transport of nutrients have been proposed [30]. While these findings may be in favor of reducing the number of layers or the thickness of the construct, certain areas, such as the femoral condyle, will require a certain thickness. Thus, a superficial osteochondral defect was necessary to embed the construct within the bone. Intraoperatively, we only had minor bleeding points in our defects, which is consistent with a superficial osteochondral defect [34]. 

Bone cyst formation has been described for full-size osteochondral defects and might be caused by fluid intrusion or bony contusion [35,36]. In contrast, in our superficial osteochondral defect model, the empty control defects did not show a significant loss of subchondral bone after 6 months. Histology did not reveal significant signs of inflammation in the area of bone loss at 6 months after implantation. Prior inflammatory responses to the PCL enforcement or the hydrogel/cell component cannot be excluded, but data of minipigs at two and 12 weeks after implantation with similar constructs provide no indication (data not shown). 

Another reason for insufficient cartilage regeneration might be the missing subchondral bone plate and stability [37]. Even though we only created a superficial osteochondral defect, the subchondral bone plate has been removed. Lack of a well-developed layer of calcified cartilage might be one of the reasons for severe subchondral bone remodeling [38], suggesting that, especially in chondral defects that affect the calcified cartilage layer, reconstruction might play a crucial role in defect healing. An approach to address this problem is to focus on reconstructing the full osteochondral unit, including the cement line and the subchondral bone plate [39]. An osteoconductive layer or addition of angiogenic or growth factors might be necessary to restore the subchondral bone plate or prevent bone lysis. To prevent an excessive reaction, a functional barrier between the subchondral bone plate and the calcified cartilage will be needed [39]. Usually, osteolysis is accompanied by macrophage activity, which might also promote cartilage degeneration [40]. An additional layer including a calcium phosphate for proper reconstruction of the subchondral bone might be crucial to prevent those bone changes and provide a sufficient foundation for zonal cartilage regeneration.

The application of cells into the constructs may be another important player in cartilage regeneration. A study by Getgood et al. in 2012 in sheep evaluated the effect of adding platelet-rich plasma or concentrated bone marrow aspirates to a biphasic osteochondral scaffold [41]. As seen in our minipigs, the group did not find differences in the mechanical properties, ICRS score or modified O’Driscoll score. McCarrel et al. in 2017 reported improved arthroscopic results of a biphasic device in osteochondral defects in an equine model when compared to microfracture [42]. Interestingly, the authors found that to some extent, microfracture showed superior to the device in regards to proteoglycan content and organized collagen, suggesting that not all studies clearly indicate superiority of biphasic constructs compared to self-healing. Similar to their findings, our study showed a superior and far more homogeneous cartilage regeneration of non-treated “self-healing” control defects, suggesting that in contrast to our hypothesis, regeneration was impaired by the constructs. Moreover, zonal design of the scaffolds did not differ from the non-zonal design concerning cartilage regeneration, and an important heterogeneity of cartilage regeneration in both treatment groups was observed.

In this study, we combined two widely used cell types in a biphasic construct that had shown promising results in vitro [18,43] and at ectopic sites in vivo [18]. The flushing out of the hydrogel containing the cells may have contributed both to the bone loss and the heterogeneous cartilage formation observed in our study. Blanke et al. were able to prove that chondrocyte transplantation may lead to a higher presence of anti-angiogenic proteins including thrombospondin and chondromodulin, which on the one hand might have a positive influence as it prevents endochondral ossification within the cartilage, but might also inhibit bone growth and, therefore, lead to more osteolysis [44]. In retrospect, an additional control group applying only hydrogel- and cell-free constructs may have contributed to determining whether the construct itself or the hydrogel/cell combination is responsible for the effects observed. Furthermore, it would have shown if an acellular construct in minipigs attracted more endogenous progenitor cells and performed better than cell-seeded constructs, as seen in a rabbit model with a PCL-based support structure for osteochondral healing [36]. 

Furthermore, later time points might be helpful as well. In a rabbit osteochondral defect, subchondral bone healing follows 4 stages that occur within the first year [45]. However, these stages have not been described for minipigs and are not fully applicable to our observations. Our 6 months’ time point, however, might not have been late enough for full subchondral bone remodeling, even in the control group. 

In conclusion, the study presented here does not contribute to the findings of other studies that suggest that biphasic scaffolds improve cartilage repair. A significant bone loss, flushing out of the hydrogel, and dislocation of the construct strongly advise against the use of the construct in the current form in a clinical setting. The study, in our opinion, reflects that while in vitro results may show applications promising for a clinical use, an achievement in vivo is not guaranteed. Despite numerous approaches to changing cartilage repair, only very few applications have made it from the bench into the patient. Furthermore, to date, no clinically available construct has shown superior to matrix-induced chondrogenesis. Assisted self-healing of the body, thus, is still part of a cartilage surgeon’s portfolio in the treatment of chondral lesions. Clinical applications of novel constructs must be supported by data clearly showing superiority over current applications. 

Our study also is in favor of a more widespread use of µCT to quantify subchondral bone loss. As we could show, even empty defects showed bone loss to some extent. To our knowledge, the studies in favor of biphasic constructs did not analyze bone loss via µCT. With regards to future clinical applications, this information, however, is crucial. 

## 4. Materials and Methods

### 4.1. Animals

The study was performed on six skeletally mature miniature pigs (Mini-Lewe), bred at the Farm for Education and Research in Ruthe, University of Veterinary Medicine Hannover, Germany, of either sex (4 male, 2 female). The average age and weight of the minipigs was 29 ± 2.6 months and 58.3 ± 14.1 kg, respectively, at the day of surgery. Animals were kept in indoor runs allowing free movement and unrestricted access to water and were fed once a day. The animal experiment was approved by the Animal Experimentation Committee Karlsruhe and was performed according to the national guidelines for animal care in accordance with European Union Directive (2010/63/EU) (Approval Code: 35-9185.81/G117/16, Approval Date: 15/09/2016). 

### 4.2. Isolation and Culture of Porcine Articular Chondrocytes

Porcine articular chondrocytes (AC) were isolated from healthy porcine knee joints (*n* = 2 donors) from slaughter pigs obtained from the local abattoir. Rinsed cartilage was cut into small pieces, digested overnight with collagenase B (1.5 mg/mL; Roche Diagnostics, Basel, Switzerland) and hyaluronidase (0.1 mg/mL; Sigma–Aldrich, St. Louis, MO, USA) and filtered through a 40 μm nylon mesh. Chondrocytes were seeded at 6000 cells/cm^2^ and expanded for 5 to 6 days in Dulbecco’s Modified Eagle’s Medium (DMEM) low glucose with L-glutamine, 10% fetal calf serum (FCS; Biochrom, Berlin, Germany), 100 U/mL penicillin, 100 µg/mL streptomycin at 37 °C and 6% CO_2_. The medium was replaced twice a week. 

### 4.3. Isolation and Culture of Porcine Mesenchymal Stromal Cells

Porcine mesenchymal stromal cells (MSC) were isolated from bone marrow obtained by bone marrow aspiration of the pelvis from 2 minipig donors. The mononuclear cell fraction was separated by density gradient centrifugation using Ficoll^®^-Paque PLUS (GE healthcare, Little Chalfont, United Kingdom), washed and seeded in expansion medium (DMEM high glucose with L-glutamine, 12.5% FCS, 100 U/mL penicillin, and 100 µg/mL streptomycin, supplemented with 4 ng/mL fibroblast growth factor-2 (FGF-2; Active Bioscience, Hamburg, Germany)). Mononuclear cells were plated at a density of 1.5 × 10^5^ cells/cm^2^ in monolayer culture and adherent MSC were subcultured for 3 passages (5 × 10^4^ cells/cm^2^) at 37 °C and 6% CO_2_.

### 4.4. Preparation of Zonal and Non-Zonal Tissue Engineering Constructs

The hydrogel was prepared by mixing thiol-endfunctionalized (non-MMP-sensitive) starPEG or starPEG–MMP-conjugates carrying MMP-sensitive peptides at every arm and maleimide functionalized heparin of a constant molar ratio of (total) starPEG to heparin of 1.5 as described before [43,46]. To obtain 20% MMP-linkers in the hydrogel, 1.2 mol (non-MMP-sensitive) starPEG and 0.3 mol starPEG–MMP-conjugates were mixed with 1 mol heparin. Hydrogel precursors were reconstituted with PBS at before mentioned molar ratios. The total solid content of the hydrogel was adjusted to be constant at 5.3% after mixing of both components. 

MSC or AC was added to the heparin solution, and hydrogels were polymerized in a disc mold enclosing a polycaprolactone (PCL) enforcement. The PCL mesh had a height of ≤1 mm and 6 mm in diameter with five layers and strand width of approximately 285 µm (Figure 1a). For non-zonal constructs, the PCL mesh was soaked with 30 µL cell-containing hydrogel (20 × 10^6^ AC/mL hydrogel) in one layer. For zonal constructs, PCL was soaked in two layers with different cell types: 15 µL hydrogel with MSC (20 × 10^6^ MSC/mL hydrogel) was cast as a bottom zone and 15 µL hydrogel with AC (20 × 10^6^ AC/mL hydrogel) as an upper layer on top. Zonal and non-zonal constructs (Figure 1a,c) were created directly before implantation and implanted by press-fit fixation into defects at the medial trochlear groove.

### 4.5. Biomechanical Testing of Zonal and Non-Zonal Constructs before Implantation 

To determine the absolute biomechanical hardness, directly after preparation zonal and non-zonal constructs (*n* = 5 per group) underwent unconfined indentation testing on a titanium plate using the mechanical testing device Digi-Test-II (Bareiss, Oberdischingen, Germany) as described before [47]. Mechanical testing defined by DIN ISO 27588 following the very low rubber hardness (VLRH) principle was applied. Each sample was analyzed twice. 

### 4.6. Orthotopic Transplantation

**Figure 7:**
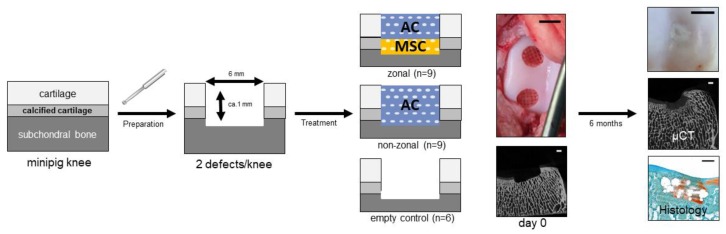
Study design. A full thickness defect was prepared in native cartilage in minipigs in vivo and treated with a zonal and a non-zonal starPEG construct containing PCL with articular chondrocytes (AC) in the top layer and MSC in the bottom layer or only AC, respectively. The outcome was compared to an empty control after 6 months of treatment via macroscopy, µCT analysis and histology. scale bar µCT images and histology: 1 mm; scale bar macroscopic images 6 mm.

For orthotopic implantation, bilateral arthrotomy was performed as described before [48]. Under general anesthesia (azaperone 5 mg/kg i.m., ketamine 15 mg/kg i.m., midazolam 0.5 mg/kg, propofol 2% i.v. to effect, inhalation anesthesia with 1–3% isoflurane in oxygen) two defects with a diameter of 6 mm and a depth of about 1 mm were created in the middle third of the medial facet of the trochlear groove of each femur (Figure 7). After marking the diameter with a biopsy punch, hyaline cartilage was detached with a curette and the calcified layer was removed using a rose head drill without causing any bleeding of the subchondral bone. Defects were treated with zonal or non-zonal hydrogel constructs or left empty as control. Constructs were carefully inserted until their surface was just under the cartilage level and held in place by press-fit fixation followed by multilayered wound closure. The animals received buprenorphine each 12 h (Buprenovet^®^, Bayer Vital GmbH, Leverkusen, Germany, 0.025 mg/kg i.m.) over 48 h postoperatively and meloxicam (Metacam^®^ 15 mg/mL, Boehringer Ingelheim Vetmedica GmbH, Ingelheim, Germany, 0.4 mg/kg p.o.) and amoxicillin (Duphamox^®^ LA, Zoetis Deutschland GmbH, Berlin, Germany, 15 mg/kg i.m.) as long as required. After four to thirteen days of postoperative analgetic and antibiotic treatment, all animals walked without any clinical signs of pain or lameness. 

Minipigs were euthanized six months postoperatively under general anesthesia by an overdose of pentobarbital (Release^®^, WDT, Garbsen, Germany). Soft tissues were removed, and the joint capsule was opened to display the defect area. After macroscopic examination and photo documentation, the trochlea was harvested, and defect areas analyzed by µCT. 

### 4.7. Macroscopic Evaluation by ICRS Score

For macroscopical evaluation of repair quality, pictures taken during explantation were assessed by three independent and blinded observers according to criteria of the ICRS score [49] given in Table 2 (best rating = 12). Scoring differences between the three independent observers where ≤2 points per defect.

### 4.8. Micro-CT Analysis

Micro-CT analysis of the defect site was performed after explantation at 6 months (*n* = 24) or directly after setting empty defects (*n* = 4) using a SkyScan 1076 in vivo X-ray microtomograph (SkyScan, Bruker-microCT, Kontich, Belgium). The following settings were used: 1.0 mm aluminum filter, source voltage 100 kV, source current 100 µA, exposure time 400 ms, voxel size 18 µm, rotation step 0.5 degree. Reconstruction of images was performed using NRecon^®^ software (version 1.6.3.2, Skyscan, Kontich, Belgium). CTAn^®^ (version 1.13.2.1, Skyscan, Kontich, Belgium) was used for calculation of the volume of bone tissue within a defined volume of interest (VOI, 8 mm × 4 mm) represented by the defect borders and 232 sectional images into the bone. The lower grey level was set at 80, and the upper grey level was set at 255. To measure the effects on bone volume/total volume in each group, one animal with four empty defects was explanted directly after surgery. Micro-CT analysis was performed to generate representative day 0 values to compare with the outcome in each group after 6 months.

### 4.9. Histology

Directly after µCT imaging porcine osteochondral samples were fixed for 72 h in 4% formaldehyde and decalcified for 13 to 15 days in Formical^®^ 2000 (StatLab, McKinney, TX, USA). Subsequently, specimens were dehydrated, PCL remnants dissolved via Xylene as intermedium, and samples embedded in paraffin. After cutting off 5 µm serial sections, slides were stained with Safranin O/Fast Green according to standard protocols. Type II collagens were stained with mouse anti-human monoclonal antibodies (MPBiomedicals, Eschwege, Germany; Jackson ImmunoResearch, Ely, United Kingdom) according to standard immune histology protocols with ImmPact Vector Red counterstaining (Vector Laboratories, Burlingame, CA, USA).

### 4.10. Modified O’Driscoll Score

For semiquantitative histomorphological evaluation of repair quality, one representative Safranin O/Fast Green stained section and one section stained for collagen type II per defect were assessed by three blinded observers according to criteria of a modified O’Driscoll score [50] given in Table 3. Scoring differences between the three independent observers where ≤2 points per defect.

## 5. Statistical Analysis

All data were normally distributed according to Shapiro-Wilk-Test. Descriptive statistics were performed for all continuous variables (VLRH and BV/TV; Figure 1b, Figure 2c,d) with mean and standard deviation (SD). Differences between groups were analyzed with one–way ANOVA and Bonferroni correction to compensate for multiple testing. Non-continuous data like histological and macroscopic scores were compared using the non-parametric Kruskal–Wallis-Test with post-hoc Mann–Whitney-U Signed-Rank Test (Figure 2b, Figure 6c). All tests were performed in accordance with two-sided testing. Differences were considered to be significant at *p* ≤ 0.05. Statistical evaluation was accomplished using SPSS (Version 25; IBM Corp. Armonk, NY, USA).

## Figures and Tables

**Table 1:** Number of defects with Safranin-O-positive regeneration tissue according to histology (Safranin O/Fast Green staining).

Group	Total	Differentiation in Hydrogel	Differentiation Connected to PCL	Non-Connected Differentiation
zonal	4/9	2	2	1
non-zonal	7/9	3	2	3
empty control	4/6	n.a. ^1^	n.a. ^1^	4

^1^ n.a. not applicable.

**Table 2:** Criteria of the ICRS macroscore used for rating of cartilage repair quality.

Characteristic	Grading	Score
Degree of defect repair	In level with surrounding cartilage	4
	75% repair of defect depth	3
	50% repair of defect depth	2
	25% repair of defect depth	1
	0% repair of defect depth	0
Integration to border zone	Complete integration with surrounding cartilage	4
	Demarcating border <1 mm	3
	3/4th of graft integrated, 1/4th with a notable border >1 mm width	2
	1/2 of graft integrated with surrounding cartilage, 1/2 with a notable border >1 mm	1
	From no contact to 1/4th of graft integrated with surrounding cartilage	0
Macroscopic appearance	Intact smooth surface	4
	Fibrillated surface	3
	Small, scattered fissures or cracks	2
	Several, small or few but large fissures	1
	Total degeneration of grafted area	0
Total		12

**Table 3:** Criteria of the used modified O´Driscoll score with a score range from 0 (worst) to 15 (best).

Characteristic	Criterion	Score Range
Nature of predominant tissue	Morphology	0–4
	Staining of matrix	0–3
Structural characteristics	Surface	0–1
	Lateral integration	0–2
	Basal integration	0–2
	Subchondral bone	0–3
Total		15

## Abbreviations

µCT	X-ray microtomography
AC	Articular cartilage
ACECM	Articular cartilage extracellular matrix
BV	Bone volume
DMEM	Dulbecco’s Modified Eagle’s Medium
FCS	Fetal calf serum
FGF	Fibroblast growth factor
ICRS	International Cartilage Repair Society
MACI	Matrix-induced autologous chondrocyte implantation
MACT	Matrix-associated autologous chondrocyte transplantation
MMP	Matrixmetalloprotease
MSC	Mesenchymal stromal cells
MWU	Mann–Whitney-U test
N	Number
n.a.	Not applicable
PCL	Polycaprolactone
PEG	Poly(ethylene glycol)
PLGA	Poly(lactid-co-glycolid)
SD	Standard deviation
TE	Tissue engineering
TV	Total volume
VLRH	Very low rubber hardness
VOI	Volume of interest
